# Potential Mediating Biomarkers underlying the Association of Body Mass Index or Waist Circumference with Blood Pressure: Results from Three Population-based Studies

**DOI:** 10.1038/s41598-017-05677-3

**Published:** 2017-07-14

**Authors:** Xiaoyan Wu, Xue Yang, Ruiqi Shan, Tianjiao Li, Tianqi Zi, Ying Li, Lixin Na, Changhao Sun

**Affiliations:** National Key Discipline, Department of Nutrition and Food Hygiene, School of Public Health, Harbin Medical University, Harbin, P. R. China

## Abstract

We conducted a comprehensive and in-depth assessment of body mass index (BMI) or waist circumference (WC) related to blood pressure (BP) and determined whether the association is mediated by the possible potential mediators in the cross-sectional survey of the Harbin Cohort Study on Diet, Nutrition and Chronic Non-communicable Diseases of 7094 participants aged 20–74 years, and validated the significant findings in the US National Health and Nutrition Examination Survey four cross-sectional cohorts (2005–2006, 2007–2008, 2009–2010, and 2011–2012) and the cohort data of the Harbin People’s Health Study (a median of 4.2 follow-up years). We observed that BMI or WC was positively associated with BP (all *P*-values < 0.0001). Mediation analyses consistently indicated that these associations were mediated mainly by insulin resistance (IR) as measured by the homeostasis model (HOMA-IR), followed by triglyceride (TG) and total cholesterol (TC), and fasting glucose (FG) in the three studies. The proportions via the mediation of insulin/HOMA-IR were 25~40%, TG and TC were 15~20%, and FG was 2~8%, respectively. These findings suggest that the mediators, insulin/insulin resistance, TG, TC, and FG, could be targeted for preventing hypertension among populations who were overweight or obesity.

## Introduction

Elevated blood pressure (BP) is a leading cause of the global burden of disease^1, 2^, and obesity has since long been demonstrated to be associated with increased risk of elevated BP in previous studies^3–6^. Although activation of the sympathetic nervous system (SNS) or renin-angiotensin-aldosterone system (RAAS) has been observed to be the key mechanism in the pathogenesis of obesity-related hypertension^7–10^, other possible mechanism in this association remains unclear. Insulin resistance, total cholesterol (TC) or triglyceride (TG) levels has been described in normotensive and hypertensive humans in some studies^11, 12^, however, no studies investigated whether these factors play a mediation role in the association of body mass index (BMI) or waist circumference (WC) with BP. Further research exploring the potential mechanism relating to these factors would provide more information for the complete elucidation of the effect of BMI or WC on BP, especially which may provide novel targets for avoiding obesity-related hypertension.

Employing a cross-sectional data from the Harbin Cohort Study on Diet, Nutrition and Chronic Non-communicable Diseases (HDNNCDS), we aimed to evaluate the association of concurrently examined the association of BMI (an indicator of overall obesity) and WC (an indicator of abdominal obesity) with BP. Specifically, employing mediation analysis^13, 14^, we tried to quantify potential mediators of biological significance which may link BMI or WC to BP. Notably, we subsequently systematically validated the findings using independent datasets from the cross-sectional survey of the US National Health and Nutrition Examination Survey (NHANES) and a cohort study of the Harbin People’s Health Study (HPHS).

## Results

**Figure 1:**
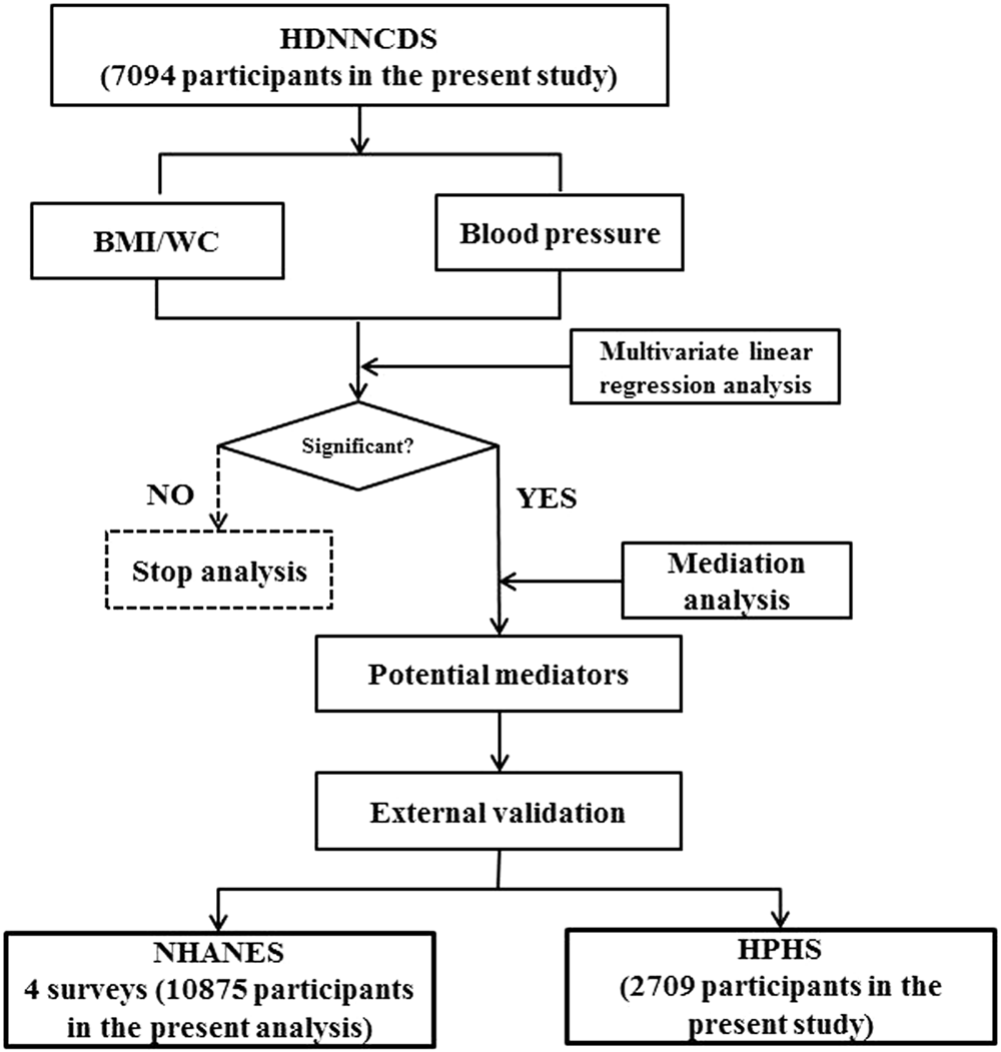
Procedure to systematically associate body mass index or waist circumference with blood pressure by potential mediators.

The analytic procedure is summarized in Fig. 1. Descriptive characteristics of the HDNNCDS, the NHANES, and the HPHS populations at baseline are shown in Table 1.

**Table 1:** Descriptive characteristics of participants at baseline in the Harbin Cohort Study on Diet, Nutrition and Chronic Non-Communicable Diseases (HDNNCDS, 2012), the US National Health and Nutrition Examination Survey (NHANES), and the Harbin People’s Health Study (HPHS, 2008–2012).

Characteristics	HDNNCDS (*n* = 7,094)	NHANES (*n* = 10,875)	HPHS (*n* = 2,709)
Age at recruitment (mean ± SD, years)	48.88 ± 10.34	45.82 ± 15.38	48.30 ± 10.46
Male (%)	35.54	19.22	30.57
Body mearsurements (mean ± SD)			
BMI (kg/m^2^)	24.63 ± 3.42	29.02 ± 6.65	24.46 ± 3.27
WC (cm)	84.84 ± 10.11	98.53 ± 16.21	82.52 ± 9.94
Lifestyle factors (%)			
Exercised regularly	45.46	12.62	57.37
Current smokers	17.31	55.05	18.31
Current drinker	35.49	74.28	38.90
Disease history (%)			
Type 2 diabetes	13.67	10.73	11.79
Coronary heart disease	12.82	2.69	17.70
Family history of hypertension	22.26	—	21.17
Blood pressure (mean ± SD, mmHg)			
Systolic blood pressure	131.66 ± 16.74	121.78 ± 17.29	121.73 ± 9.66
Diastolic blood pressure	80.28 ± 8.28	70.81 ± 12.19	77.33 ± 5.09
Total energy intake (mean ± SD, kcal/day)	2316.47 ± 744.03	2069.97 ± 763.24	2176.04 ± 699.77
Lipid profiles (mean ± SD)			
TG (mmol/L)	1.73 ± 1.82	1.49 ± 1.21	1.68 ± 1.47
TC (mmol/L)	5.12 ± 1.03	5.08 ± 1.07	4.87 ± 0.97
HDL (mmol/L)	1.27 ± 0.33	1.36 ± 0.40	1.29 ± 0.34
LDL (mmol/L)	3.01 ± 0.85	3.00 ± 0.91	2.85 ± 1.00
Glucose (mean ± SD)			
FG (mmol/L)	4.92 ± 1.65	5.87 ± 1.79	5.07 ± 2.79
PG (mmol/L)	6.24 ± 2.86	6.43 ± 2.81	6.34 ± 3.64
FI (uU/L)	8.47 ± 7.50	13.61 ± 15.17	8.37 ± 9.01
PI (uU/L)	42.68 ± 46.87	—	32.83 ± 27.09
HOMA-IR	1.93 ± 2.36	3.75 ± 5.20	1.95 ± 3.05
HOMA-B	268.75 ± 891.83	130.80 ± 145.20	178.37 ± 242.15

Abbreviation: SD, standard deviation; BMI, body mass index; WC, waist circumference; TG, triglyceride; TC, total cholesterol; HDL, high density lipoprotein; LDL, low density lipoprotein; FG, fasting glucose; PG, 2-h postprandial glucose; FI, fasting insulin; PI, 2-h postprandial insulin.

### Associations between BMI or WC and BP

After adjusted for potential confounders, BMI or WC was significantly positively associated with systolic BP or diastolic BP in the HDNNCDS, the NHANES, and the HPHS (all *P*-values < 0.0001) (Table 2).

**Table 2:** Adjusted estimated differences in systolic or diastolic blood pressure associated with BMI or WC by 1 SD in the Harbin Cohort Study on Diet, Nutrition and Chronic Non-Communicable Diseases (HDNNCDS, 2012), the Harbin People’s Health Study (HPHS, 2008–2012), and the US National Health and Nutrition Examination Survey (NHANES).

	HDNNCDS (*n* = 7,094)		NHANES (*n* = 10,875)		HPHS (*n* = 2,709)	
	Difference–mm Hg (95% CI)	*P*-value	Difference–mm Hg(95% CI)	*P*-value	Difference–mm Hg(95% CI)	*P*-value
**SBP**						
BMI(kg/m^2^)						
Model1	2.62 (2.22,3.03)	<0.0001	2.39 (2.07,2.71)	<0.0001	1.34 (0.73,1.96)	<0.0001
Model2	2.16 (1.54,2.78)	<0.0001	2.22 (1.88,2.55)	<0.0001	1.23 (0.60,1.86)	0.0001
WC (cm)						
Model1	3.42 (2.99,3.84)	<0.0001	2.11 (1.79,2.44)	<0.0001	1.50 (0.86,2.15)	<0.0001
Model2	2.73 (2.06,3.40)	<0.0001	1.92 (1.58,2.26)	<0.0001	1.43 (0.75,2.10)	<0.0001
**DBP**						
BMI (kg/m^2^)						
Model1	1.19 (0.98,3.84)	<0.0001	1.53 (1.28,1.78)	<0.0001	0.75 (0.41,1.09)	<0.0001
Model2	1.11 (0.78,1.44)	<0.0001	1.72 (1.46,1.97)	<0.0001	0.74 (0.39,1.08)	<0.0001
WC (cm)						
Model1	1.37 (1.14,1.59)	<0.0001	1.44 (1.18,1.69)	<0.0001	0.89 (0.54,1.24)	<0.0001
Model2	1.20 (0.85,1.56)	<0.0001	1.66 (1.40,1.92)	<0.0001	0.82 (0.45,1.18)	<0.0001

Abbreviation: BMI, body mass index; WC, waist circumference; SBP, systolic blood pressure; DBP, diastolic blood pressure. Model1: adjusted for age and sex. Model2: adjusted for age, sex, current smoking, current drinking, exercise regularly, type 2 diabetes, cardiovascular disease, family history of hypertension, and total energy intake in the HPHS and HDNNCDS; Adjusted for age, sex, ethnicity, exercise regularly, current smoking, current drinking, type 2 diabetes, cardiovascular disease, and total energy intake in the NHANES.

### Mediation analysis

In the HDNNCDS, we found that TG, TC, FG, PG, FI, and HOMA-IR were the potential mediators between BMI or WC and systolic BP or diastolic BP. Additionally, HOMA-B was the potential mediator between BMI or WC and systolic BP, and PI was the potential mediator between WC and diastolic BP. Among these mediators, the proportions via mediation by FI, HOMA-IR, and TG were the top three ones which totally accounted for more than 30% of the total effect, they were 12.0%, 10.6%, and 9% between BMI and systolic BP, 10.2%, 9.1%, and 6.6% between WC and systolic BP, 19.8%, 16.9%, and 13.4% between BMI and diastolic BP, 19.7%, 15.8%, and 11.4% between WC and diastolic BP, respectively. The proportions via mediation by other mediators were ranged from 1.8% to 4.8% (Table 3).

**Table 3:** Mediation analysis between BMI/WC and blood pressure in the Harbin Cohort Study on Diet, Nutrition and Chronic Non-Communicable Diseases (HDNNCDS, 2012).

Mediators (by 1 SD)	Outcomes	BMI^*^				WC^*^			
		Total effect, estimate (95% CI)	Proportion via mediation, estimate (95% CI)	Sensitivity analysis		Total effect, estimate (95% CI)	Proportion via mediation, estimate (95% CI)	Sensitivity analysis	
				R^2*^	${\tilde{{\rm{R}}}}^{2}$			R^2*^	${\tilde{{\rm{R}}}}^{2}$
TG	SBP	2.496 (2.013,2.890)	0.090 (0.057,0.137)	0.01	0.0073	3.254 (2.939,2.758)	0.066 (0.040,0.090)	0.01	0.0072
	DBP	1.155 (0.879,1.350)	0.134 (0.090,0.184)	0.01	0.0081	1.345 (1.118,1.584)	0.114 (0.087,0.155)	0.01	0.0081
TC	SBP	2.496 (2.142,2.904)	0.018 (0.006,0.033)	0.01	0.0077	3.254 (2.809,3.716)	0.018 (0.009,0.034)	0.01	0.0076
	DBP	1.155 (0.918,1.346)	0.021 (0.006,0.040)	0.01	0.0086	1.345 (1.128,1.568)	0.024 (0.009,0.039)	0.01	0.0086
HDL	SBP	2.494 (2.150,2.886)	−0.013 (−0.043,0.028)	NA	NA	3.250 (2.899,3.767)	−0.013 (−0.042,0.019)	NA	NA
	DBP	1.153 (0.092,1.360)	0.018 (−0.031,0.063)	NA	NA	1.344 (1.137,1.479)	0.016 (−0.023,0.058)	NA	NA
LDL	SBP	2.491 (1.965,2.864)	0.014 (−0.001,0.032)	NA	NA	3.251 (2.838,3.647)	0.008 (−0.0003,0.020)	NA	NA
	DBP	1.149 (0.959,1.378)	0.012 (−0.006,0.030)	NA	NA	1.340 (1.135,1.553)	0.008 (−0.004,0.023)	NA	NA
FG	SBP	2.491 (2.052,0.822)	0.048 (0.031,0.070)	0.01	0.0049	3.252 (2.856,3.638)	0.043 (0.026,0.057)	0.01	0.0048
	DBP	1.154 (0.971,1.429)	0.040 (0.021,0.065)	0.01	0.0055	1.344 (1.124,1.568)	0.040 (0.022,0.065)	0.01	0.0055
PG	SBP	2.561 (2.117,3.041)	0.045 (0.019,0.079)	0.01	0.0052	3.277 (2.866,3.815)	0.034 (0.016,0.051)	0.01	0.0051
	DBP	1.218 (0.959,1.469)	0.039 (0.017,0.066)	0	0	1.435 (1.218,1.646)	0.033 (0.011,0.064)	0	0
FI	SBP	2.642 (2.263,3.184)	0.120 (0.048,0.181)	0.01	0.0074	3.072 (2.058,3.670)	0.102 (0.032,0.163)	0.01	0.0073
	DBP	1.114 (0.812,1.467)	0.198 (0.125,0.316)	0.01	0.0082	1.211 (0.917,1.495)	0.197 (0.117,0.298)	0.01	0.0082
PI	SBP	2.723 (2.273,3.246)	0.040 (−0.008,0.092)	NA	NA	2.908 (2.300,3.400)	0.041 (0.008,0.099)	0	0
	DBP	1.213 (0.967,1.556)	0.051 (−0.006,0.134)	NA	NA	1.305 (0.977,1.614)	0.052 (−0.001,0.123)	NA	NA
HOMA-IR	SBP	2.647 (2.055,3.202)	0.106 (0.064,0.152)	0.01	0.0075	3.070 (2.584,3.553)	0.091 (0.045,0.150)	0.01	0.0075
	DBP	1.115 (0.866,1.406)	0.169 (0.098,0.262)	0.01	0.0083	1.224 (0.961,1.567)	0.158 (0.101,0.222)	0.01	0.0084
HOMA-B	SBP	2.509 (2.011,3.000)	−0.027 (−0.060,−0.006)	0	0	2.937 (2.488,3.562)	−0.025 (−0.048,−0.004)	0	0
	DBP	1.098 (0.829,1.344)	0.002 (−0.033,0.029)	NA	NA	1.177 (0.881,1.451)	0.003 (−0.030,0.040)	NA	NA

Abbreviation: BMI, body mass index; WC, waist circumference; SBP, systolic blood pressure; DBP, diastolic blood pressure; TG, triglyceride; TC, total cholesterol; HDL, high-density lipoprotein; LDL, low-density lipoprotein; FG, fasting glucose; PG, 2-h postprandial glucose; FI, fasting insulin; PI, 2-h postprandial insulin.

*Adjusted for age, sex, current smoking, current drinking, exercise regularly, type 2 diabetes, cardiovascular disease, family history of hypertension, and total energy intake. R^2*^, the proportion of residual variances and ${\mathop{{\rm{R}}}\limits^{\sim }}^{2}$, the proportion of original variances that were explained by the omitted confounding.

In the validation analysis, the potential mediators of TG, TC, FG, and HOMA-IR were externally validated in the cross-sectional survey of the NHANES and the cohort data of the HPHS (Table 4). In addition, PG and FI were validated in the NHANES; PI was validated in the HPHS. But HOMA-B was not validated in the two studies and PI could not be validated in the NHANES since it was not measured in this study.

**Table 4:** Mediation analysis between BMI or WC and blood pressure in the US National Health and Nutrition Examination Survey (NHANES) and in the Harbin People’s Health Study (HPHS, 2008–2012).

Mediators (by 1 SD)	Outcomes	BMI				WC			
		Total effect, estimate (95% CI)	Proportion via mediation, estimate (95% CI)	Sensitivity analysis		Total effect, estimate (95% CI)	Proportion via mediation, estimate (95% CI)	Sensitivity analysis	
				R^2*^	${\tilde{{\rm{R}}}}^{2}$			R^2*^	${\tilde{{\rm{R}}}}^{2}$
**NHANES** ^**a**^									
TG	SBP	2.311 (1.851,2.809)	0.123 (0.070,0.182)	0.01	0.0070	2.014 (1.577,2.506)	0.161 (0.095,0.241)	0.01	0.0071
	DBP	1.763 (1.443,2.098)	0.157 (0.101,0.223)	0.01	0.0083	1.785 (1.449,2.155)	0.168 (0.115,0.227)	0.01	0.0083
TC	SBP	2.221 (1.851,2.541)	0.024 (0.011,0.038)	0.01	0.0074	1.906 (1.599,2.170)	0.029 (0.014,0.049)	0.01	0.0075
	DBP	1.749 (1.437,2.061)	0.036 (0.014,0.056)	0.01	0.0087	1.677 (1.449,1.905)	0.039 (0.019,0.066)	0.01	0.0087
FG	SBP	2.330 (1.942,2.827)	0.068 (0.032,0.097)	0.01	0.0054	2.039 (1.471,2.508)	0.085 (0.028,0.138)	0.01	0.0054
	DBP	1.739 (1.398,2.087)	0.057 (0.021,0.096)	0	0	1.761 (1.238,2.101)	0.058 (0.023,0.106)	0	0
PG	SBP	2.615 (1.999,3.037)	0.205 (0.145,0.277)	0.01	0.0067	2.275 (1.732,2.719)	0.259 (0.180,0.374)	0.01	0.0067
	DBP	1.886 (1.467,2.260)	0.077 (0.022,0.135)	0	0	1.800 (1.422,2.211)	0.187 (0.084,0.327)	0	0
FI	SBP	2.394 (1.983,2.866)	0.144 (0.056,0.245)	0	0	2.143 (1.769,2.566)	0.202 (0.066,0.330)	0	0
	DBP	1.761 (1.500,2.139)	0.193 (0.07,0.320)	0	0	1.800 (1.422,0.327)	0.187 (0.084,0.327)	0	0
HOMA-IR	SBP	2.388 (1.826,2.886)	0.119 (0.034,0.244)	0	0	2.137 (1.678,2.598)	0.165 (0.038,0.298)	0	0
	DBP	1.761 (1.365,2.128)	0.189 (0.070,0.339)	0.01	0.0073	1.799 (1.439,2.265)	0.186 (0.084,0.286)	0.01	0.0072
HOMA-B	SBP	2.413 (1.885,2.912)	0.015 (−0.071,0.111)	NA	NA	2.157 (1.723,2.624)	0.043 (−0.059,0.155)	NA	NA
	DBP	1.768 (1.399,2.085)	0.054 (−0.047,0.176)	NA	NA	1.808 (1.409,2.229)	0.052 (−0.047,0.156)	NA	NA
**HPHS** ^**b**^									
TG	SBP	1.306 (0.667,1.806)	0.114 (0.046,0.306)	0.010	0.0080	1.422 (0.754–1.949)	0.113 (0.033,0.324)	0.010	0.0077
	DBP	0.728 (0.455–1.025)	0.005 (−0.034,0.062)	NA	NA	0.920 (0.469–1.298)	0.162 (0.069,0.306)	0.010	0.0081
TC	SBP	1.297 (0.886,1.836)	0.047 (−0.002,0.141)	NA	NA	1.414 (0.774–2.004)	0.051 (0.004,0.124)	0.008	0.0070
	DBP	0.702 (0.337–1.055)	0.019 (−0.042,0.091)	NA	NA	0.893 (0.491–1.222)	0.016 (−0.028,0.077)	NA	NA
FG	SBP	1.299 (0.678–1.903)	0.040 (0.001,0.103)	0.010	0.0060	1.416 (0.699–2.082)	0.046 (0.004,0.102)	0.010	0.0060
	DBP	0.717 (0.380–1.077)	0.023 (−0.0063,0.082)	NA	NA	0.911 (0.517–1.193)	0.021 (−0.007,0.076)	NA	NA
PG	SBP	1.172 (0.640–1.737)	0.002 (−0.051,0.076)	NA	NA	1.307 (0.681–2.068)	−0.006 (−0.108,0.096)	NA	NA
	DBP	0.695 (0.357–1.007)	0.005 (−0.059,0.060)	NA	NA	0.868 (0.544–1.223)	−0.004 (−0.087,0.062)	NA	NA
FI	SBP	2.061 (1.432–2.749)	0.103 (0.039,0.213)	0.010	0.0071	1.918 (1.134–2.784)	0.145 (0.042,0.298)	0.007	0.0071
	DBP	1.585 (1.199–2.018)	0.151 (0.037,0.351)	0.010	0.0081	2.616 (1.805–2.998)	0.160 (0.054,0.311)	0.010	0.0080
PI	SBP	0.999 (0.295–1.621)	0.098 (−0.058,0.631)	NA	NA	1.180 (0.327–2.047)	0.292 (0.103,0.902)	0.010	0.0080
	DBP	0.641 (0.202–1.083)	0.088 (−0.043,0.428)	NA	NA	0.824 (0.518–1.277)	0.075 (−0.051,0.270)	NA	NA
HOMA-IR	SBP	2.346 (2.011–3.108)	0.108 (0.043,0.281)	0.010	0.0070	2.956 (2.494–3.419)	0.081 (0.040,0.146)	0	0.0071
	DBP	1.121 (0.892–1.609)	0.111 (0.041,0.298)	0.010	0.0080	1.209 (0.906–1.553)	0.143 (0.081,0.192)	0.007	0.0080
HOMA-B	SBP	1.049 (0.348–1.947)	−0.058 (−0.243,0.044)	NA	NA	0.846 (−0.044–1.629)	−0.069 (−0.683,0.117)	NA	NA
	DBP	0.530 (0.103–1.018)	−0.001 (−0.177,0.205)	NA	NA	0.054 (0.016–1.031)	0.0001 (−0.0152,0.184)	NA	NA

Abbreviation: BMI, body mass index; WC, waist circumference; SBP, systolic blood pressure; DBP, diastolic blood pressure; TG, triglyceride; TC, total cholesterol; FG, fasting glucose; PG, 2-h postprandial glucose; FI, fasting insulin. ^a^ Adjusted for age, sex, ethnicity, exercise regularly, current smoking, current drinking, diabetes mellitus, cardiovascular disease, and total energy intake. ^b^Adjusted for age, sex, current smoking, current drinking, exercise regularly, type 2 diabetes, cardiovascular disease, family history of hypertension, and total energy intake. R^2*^, the proportion of residual variances and ${\tilde{{\rm{R}}}}^{2}$, the proportion of original variances that were explained by the omitted confounding.

In the mediation analysis of the association between BMI or WC and IR (indicated by HOMA-IR), TG, TC, FG accounted for 5.4–11.3%, 0.3–2.0%, and 9.4–16.7% of the total effect on IR due to BMI or WC in the HDNNCDS, NHANES, or HPHS, respectively (Table 5).

**Table 5:** Effect of BMI or WC on insulin resistance with mediation of established biomarkers in the Harbin Cohort Study on Diet, Nutrition and Chronic Non-Communicable Diseases (HDNNCDS, 2010–2012), the US National Health and Nutrition Examination Survey (NHANES) and in the Harbin People’s Health Study (HPHS, 2008–2012)

Mediators (by 1 SD)	BMI				WC			
	Total effect, estimate (95% CI)	Proportion via mediation, estimate (95% CI)	Sensitivity analysis		Total effect, estimate (95% CI)	Proportion via mediation, estimate (95% CI)	Sensitivity analysis	
			R^2*^	${\tilde{{\rm{R}}}}^{2}$			R^2*^	${\tilde{{\rm{R}}}}^{2}$
**HDNNCDS** ^**a**^								
TG	0.106 (0.092,0.121)	0.074 (0.049,0.102)	0.01	0.0082	0.107 (0.091,0.122)	0.082 (0.055,0.114)	0.01	0.0082
TC	0.106 (0.092,0.119)	0.006 (−0.003,0.015)	NA	NA	0.106 (0.091,0.124)	0.001 (0.009,0.023)	0.01	0.0087
FG	0.106 (0.092,0.120)	0.094 (0.051,0.133)	0.09	0.0469	0.107 (0.089,0.122)	0.167 (0.069,0.154)	0.09	0.0476
**NHANES** ^**b**^								
TG	0.298 (0.276,0.321)	0.054 (0.043,0.071)	0.04	0.0277	0.295 (0.268,0.317)	0.058 (0.044,0.076)	0.04	0.0273
TC	0.298 (0.271,0.318)	0.020 (0.011,0.028)	0.01	0.0071	0.295 (0.275,0.319)	0.018 (0.011,0.026)	0.01	0.0071
FG	0.298 (0.274,0.322)	0.107 (0.079,0.138)	0.16	0.0748	0.295 (0.273,0.316)	0.105 (0.081,0.134)	0.16	0.0740
**HPHS** ^**a**^								
TG	0.166 (0.118,0.215)	0.106 (0.042,0.187)	0.01	0.0072	0.166 (0.111,0.218)	0.113 (0.051,0.200)	0.04	0.0293
TC	0.171 (0.119,0.221)	0.010 (−0.008,0.038)	NA	NA	0.171 (0.126,0.220)	0.003 (0.008,0.021)	0.01	0.0083
FG	0.168 (0.166,0.218)	0.102 (0.029,0.198)	0.09	0.0481	0.168 (0.115,0.224)	0.117 (0.024,0.227)	0.09	0.0488

Abbreviation: BMI, body mass index; WC, waist circumference; SBP, systolic blood pressure; DBP, diastolic blood pressure; TG, triglyceride; TC, total cholesterol; FG, fasting glucose; PG, 2-h postprandial glucose; FI, fasting insulin. ^a^Adjusted for age, sex, current smoking, current drinking, exercise regularly, type 2 diabetes, cardiovascular disease, family history of hypertension, and total energy intake. ^b^ Adjusted for age, sex, ethnicity, exercise regularly, current smoking, current drinking, diabetes mellitus, cardiovascular disease, and total energy intake. R^2*^, the proportion of residual variances and ${\tilde{{\rm{R}}}}^{2}$, the proportion of original variances that were explained by the omitted confounding.

The robustness of all mediation results was supported by sensitivity analyses that showed omitted confounding effect could explain less than 10 percent of variances.

### Cross-lagged path analysis

**Figure 2:**
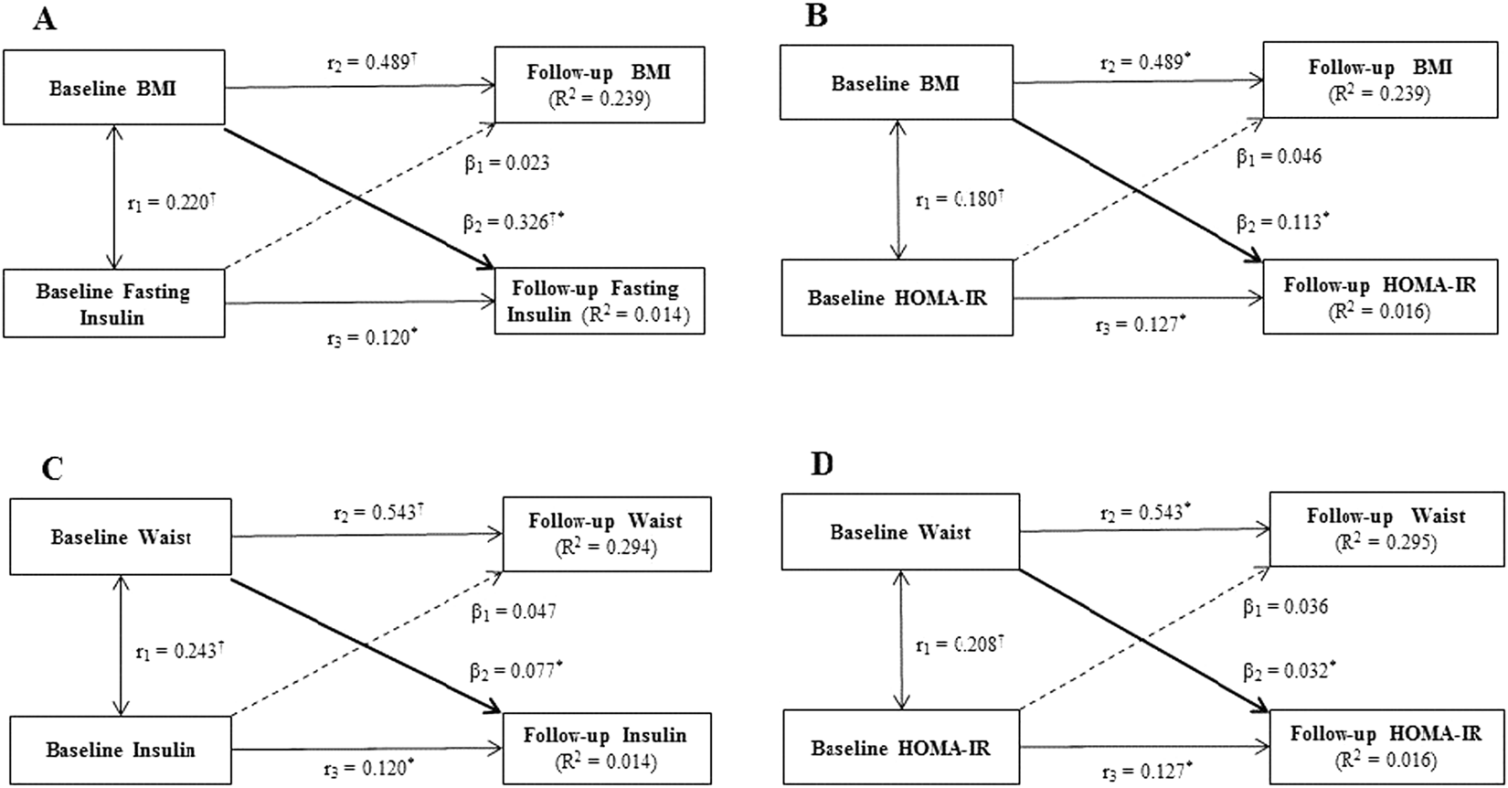
Cross-lagged path analysis of BMI or WC and insulin or HOMA-IR in the Harbin People Health Study, adjusted for age, sex, and follow-up years. β1 and β2 are cross-lagged path coefficients; *r*1 is synchronous correlations; *r*2 and *r*3 are tracking correlations; R^2^ is variance explained. Coefficients different from 0: *P < 0.01, ^†^ *P* < 0.001 for difference between β1 and β2.

Figure 2 presents cross-lagged path analysis of BMI or WC and insulin or HOMA-IR in the HPHS, which indicated that increased BMI or WC preceded hyperinsulinemia. After adjusting for age, sex, and follow-up years, the path coefficient from baseline BMI to follow-up insulin (β_2_ = 0.326) was significantly greater than the path coefficient from baseline insulin to follow-up BMI (β_1_ = 0.0.023), with *P* = 0.001 for difference between β_1_ and β_2_. Autocorrelation also known as tracking correlation of BMI (*r*_2_) were significantly greater than that of insulin (*r*_3_). The variance (*R* ^2^) of follow-up BMI explained by baseline predictors was greater than that of follow-up insulin (Fig. 2(A)). And the path coefficients from baseline BMI to follow-up HOMA-IR, baseline WC to follow-up insulin, baseline WC to follow-up HOMA-IR were 0.113 (*P* < 0.0001), 0.077 (*P* = 0.020), and 0.032 (*P* = 0.001), respectively, however, the path coefficients from baseline HOMA-IR to follow-up BMI, baseline insulin to follow-up WC, baseline HOMA-IR to follow-up WC were not statistically significant (Fig. 2(B, C, and D)). These results provided stronger evidence for the results in the mediation analysis in the present study.

## Discussion

In the present study, we confirmed that BMI or WC was significantly positively associated with BP. By using mediation analysis, we identified insulin concentrations (FI and PI) and HOMA-IR mediated a considerable amount of the total effect of BMI or WC on BP. This effect was further mediated by TG, TC, and FG. To the best of our knowledge, this study is the first to directly provide quantifiable mechanistic evidence linking BMI or WC to BP in the three independent population-based studies.

Significant association of BMI or WC with BP has been observed consistently in different populations^4, 5, 15, 16^. Doll *et al*. found that systolic BP and diastolic BP were increased over the whole variation range of BMI and WC among populations across developed and developing countries (from the main Seychelles island (Mahe) and two Swiss regions (Vaud-Fribourg and Ticino)), in which a gain of 1.7 kg/m^2^ in BMI or of 4.5 cm in WC corresponded to an elevation of 1 mmHg in systolic BP for men, and the corresponding figures were 1.25 kg/m^2^ and 2.5 cm for women, respectively^5^. Bovet *et al*. observed that BMI was positively associated with BP in a cross-sectional survey of the entire population in five branches of Dar es Salaam (1.01 and 0.91 mmHg systolic BP per 1 kg/ m^2^ BMI in men and women, respectively)^4^. In agreement with these findings, our results showed similar positive association of BMI or WC with BP in a cross-sectional survey of the HDNNCDS, and it is particularly validated in American population in the cross-sectional surveys of the NHANES and an independent Chinese cohort of the HPHS in the present study.

Although results reported in an appreciable number of studies have supported these associations, the potential mechanisms remain to be an area of research. In addition, proximal mediators of the effect are more relevant to targeted elevated BP prevention among obesity persons yet this topic remains understudied. Mediation analysis was first proposed and has been prominent statistical analysis in psychological research^17^. Under appropriate causal structures justified by substantive scientific knowledge, mediation analysis addresses directly the questions of how and why the specific exposure and outcome are related by the intermediate factor through measuring its contribution to the effect of the exposure on the outcome. By using this method, we evaluated the potential mediation effects of possible mediators of BP and resulted in the observations that several mediators may be partially determined by BMI or WC and also predict BP.

The link between obesity and the development of insulin resistance has been well documented. The values of the FI and HOMA-IR have been observed significantly higher in the obese subjects in comparison with the subjects with normal weight^18^, and weight loss/gain correlates closely with a decrease/an increase in insulin sensitivity, respectively^19^. It has been proposed that obese individuals develop resistance to the cellular actions of insulin, characterized by an impaired ability of insulin to inhibit glucose output from the liver and to promote glucose uptake in fat and muscle^20, 21^. Meanwhile, an association between insulin metabolism/resistance and BP has been reported in previous studies as well. Insulin can cause vasodilation through increased NO production, any reduction in the functionality of the peptide will obviously have adverse effects on blood pressure. Additionally, insulin resistance causes increased blood flow to skeletal muscles to compensate for the reduced glucose delivery, which in turn also increases BP^22^. Insulin concentrations have been observed to be significantly higher in adult patients with hypertension and borderline hypertension than in normotensive control patients, no matter whether insulin is measured in the fasting state or in response to the oral glucose tolerance test^2, 23^. However, study that provided whether insulin/insulin resistance is playing a role in linking BMI or WC with BP is limited. We performed mediation analysis to examine this association. Our data appear to indicate that insulin (FI and PI) and insulin resistance (HOMA-IR) may have a significant mediating effect on the association between BMI or WC and BP, which accounted for about 25~40% of the total effect of this association. This finding adds further support to previous speculations that the association between BMI or WC and BP might be mediated by insulin/insulin resistance.

In addition, obesity has been speculated to be the main cause of the metabolic syndrome (MetS), and dyslipidemia is an important feature in MetS^24^. As expected, epidemiological studies have observed that both TC and TG keep increasing steadily with BMI and WC^25–27^. At the same time, TC and TG are usually associated with increased BP levels, and the increases in TC and TG level with BP were observed to be greater in overweight than in lean subjects^28^. This suggests that body mass in itself or factors associated with body mass are related to concomitant elevations of BP and blood lipids. In the present study, we found that TC and TG were mediators linking BMI or WC with BP, which explained approximately 15~20% of the total effect of this association. Moreover, BMI or WC and FG have been reported to be positively correlated in previous studies^29, 30^, and the association between elevated FG and risk of the development of hypertension has been described in some studies^31^. In the present study, we observed that FG was also a mediator linking BMI or WC and BP, which explained a small but significant amount of the total effect of BMI or WC on BP.

The strengths of our study include its base in three independent population-based surveys among Chinese and American populations and hence the ability to validate the findings in these studies, in which there is one cohort study with a high follow-up rate. Specifically, we used the novel counterfactual model-based mediation analysis rather than the traditional method that compares differences of the regression coefficients between the models with and without the mediators. Our work has limitations. First, although we adjusted for confounders, we cannot exclude the possibility of residual confounding. Second, the SNS and RAAS have also suggested being etiologically relevant in obesity related hypertension; however, we did not measure them in the present study. Thus, we cannot discuss their relevance to increased BMI or WC induced BP elevation. Third, mediating effects may not be reliable detected in the two present cross-sectional studies, but the results from the present follow-up data of the HPHS adds further support of the mediating effects of the potential mediators.

## Conclusions

The present study confirmed that BMI or WC was consistently positively associated with BP, particularly across three different populations. In addition, the total effect of the association between BMI or WC and BP was found to be mainly mediated by insulin/insulin resistance, which is further explained by TG, TC and FG. These validated data provide quantifiable mechanistic evidence linking BMI or WC to BP, which may provide targets for avoiding obesity-related hypertension.

## Methods

### Study Populations

The HDNNCDS and the HPHS methods have been previously described in detail^32^.The HDNNCDS was launched in 2010 by the national key discipline, department of nutrition and food hygiene at Harbin Medical University, which recruited a total of 9734 people aged 20–74 years and the baseline survey was finished in 2012. After excluding those who were taken medications for hypertension (n = 1779), those who reported extreme values for total energy intake (>4500 or < 500 kcal/day, n = 368), and those who had missing information on BMI or WC (n = 493), 7094 participants were included in the present study. The HPHS recruited 8940 people aged 20–74 years in 2008. After finishing the baseline survey, 4515 members (about 50.5% of total participants) were randomly selected to participate in the follow-up surveys due to financial limit for this study. In 2012, 4158 participants finished the first in-person follow-up survey with a response rate of 92.1%. After excluding those who had hypertension at baseline survey (*n* = 1492), those who reported extreme values for total energy intake (>4500 or < 500 kcal/day, *n* = 177), and those who had missing informatio*n* on BMI or WC (*n* = 137), 2709 participants were included in the present study. In both the HDNNCDS and the HPHS, detailed in-person interviews were administered by trained personnel using a structured questionnaire to collect information on demographic characteristics, dietary habits, and lifestyles and physical condition at baseline survey. Weight and height were measured with participants standing without shoes and wearing light clothing at baseline recruitment. BMI (kg/m^2^) was calculated as weight (kg) divided by the square of the height in meters (m^2^). The seated blood pressures of the subjects were measured on the right arm after 5 min of rest to the nearest 2 mmHg by using an electronic blood pressure monitor (OMRON HEM-7112), the mean of the two measurements was recorded in the HDNNCDS, and the mean BP at follow-up survey in the HPHS were used in the present study. Serum TC, TG, high density lipoprotein (HDL), and low density lipoprotein (LDL) were determined using an automatic biochemical analyzer (Hitachi 7100, Japan). An oral glucose tolerance test (OGTT) was carried out according to the World Health Organization (WHO) guidelines^33^. Serum insulin was measured with an auto-analyzer using commercial kits (Centaur, Bayer Corporation, Bayer Leverkusen, Germany). Homeostasis model assessment of insulin resistance (HOMA-IR) was calculated according to the formula: FG (mmol/L) × Fasting insulin (FI) (mIU/L)/22.5, and HOMA-B was calculated with the formula: 20 × FI (mIU/L)/FG (mmol/L) −3.5^34^.

The HDNNCDS and the HPHS were approved by the institutional ethics review board of the Harbin Medical University and were conducted in accordance with the guidelines of the Declaration of Helsinki. Written informed consent was provided by all participants.

NHANES is a cross-sectional, biannual, representative health survey of the United States population^35^. We used data from four surveys (2005–2006, 2007–2008, 2009–2010, and 2011–2012). 24-hour food recall questionnaire were administered using the United States Department of Agriculture (USDA) and US Department of Health and Human Services (DHHS) food recall questionnaire. Self-reported data were also collected for diabetes mellitus, coronary disease status, current drinking, and current smoking. Height, weight, and 3 to 4 seated systolic blood pressure (systolic BP) and diastolic blood pressure (diastolic BP) measurements were also administered. The mean of the BP values (3–4 measurements) were used for analysis. Serum lipids profiles (including TC, TG, HDL, and LDL), OGTT, and FI were also measured. We limited the study sample to adults who were aged 20 to 74 years, were not pregnant among females, did not take extreme values for total energy intake (<500 kcal/day or >4500 kcal/day), and did not have missing or unknown information on BMI, WC, BP, current drinking, education, coronary heart disease, and type 2 diabetes for consistency across surveys and comparable to the participants in the HDNNCDS and the HPHS. At last, 10875 subjects were analyzed in the present study.

### Statistical Analyses

Selected baseline characteristics were presented with mean ± standard deviation (SD) for continuous variables and percentage for categorical variables. Linear regression models were employed to evaluate the effect of BMI or WC per SD difference on BP. The two main models were as follows: Model 1 was adjusted for age at study recruitment (years) and sex (male/female). Model 2 was adjusted for age at study recruitment (years), sex (male/female), current smoking (yes/no), current drinking (yes/no), exercise regularly (yes/no), type 2 diabetes (yes/no), cardiovascular disease (yes/no), and total energy intake (kcal/day) in the NHANES and additionally adjusted for family history of hypertension (yes/no) in the HDNNCDS and HPHS. Mediation analysis was performed to evaluate the role of fasting glucose (FG), 2-h postprandial glucose (PG), FI, 2-h postprandial insulin (PI), HOMA-IR, HOMA-B, TC, TG, HDL, and LDL at baseline as potential mediators of the association of BMI or WC at baseline with BP. Mediation effect was evaluated by the degree of attenuation of the per SD increment of BMI or WC effect by further adjusting for the potential mediators in the linear regression models. The proportion of mediating effects was calculated in the risk difference scale, and the 95% CIs of the portions of effects were obtained via bootstrapping^36^.

In addition, the relationship between obesity and insulin has been suggested to be reciprocal^37^ and thus, the causal sequence between them should be figured out in longitudinal cohort in order to explain the results in the mediation analysis. The cross-lagged panel analysis is a form of path analysis that simultaneously examines reciprocal, longitudinal relationships among a set of intercorrelated variables^38, 39^. It was used to analyze data to provide stronger evidence for a temporal relationship between BMI or WC and insulin or HOMA-IR in longitudinal cohort of the HPHS. Pearson correlation coefficients of the Z-transformed quantitative variables of BMI or WC and insulin or HOMA-IR at baseline and follow-up were calculated, with adjustment for follow-up years.

All analyses were performed by using R version 3.0.3 (http://www.r-project.org/) and a two-sided *P*-value < 0.05 was considered statistically significant.
